# Acromegaly

**Published:** 1899-02

**Authors:** 


Acromegaly. An Essay to which was awarded the Boylston Prize of Harvard University for the year 1898. By Guy Hinsdale, A. M., M. D. Fellow of the College of Physicians of Philadelphia, and of the American Academy of Medicine; Member of the American Neurological and Climatological Associations; Assistant Physician to the Orthopedic Hospital and Infirmary for Nervous Diseases, and to the Presbyterian Hospital, Philadelphia. Detroit: Reprinted from *Medicine*, William M. Warren, Publisher. 1898.

The publication of this essay in book form will be received with pleasure by all workers in neurology and those who strive to keep abreast of general medical literature. It is a subject interesting to the osteologist, myologist, embryologist, as well as the neurologist, and Dr. Hinsdale has succeeded admirably in his prize-winning presentation of this disease. The essay is based on an analysis of 130 cases, which have been studied as originally published, with a bibliography of 377 references. Many full page illustrations are embodied in the essay and the whole subject is treated in an interesting and intelligible manner.

The essay was published in serial form in *Medicine* and is now put into book form in an attractive dress.

